# The effect of self-rated health, subjective socioeconomic status, social capital, and physical activity on life satisfaction: a cross-sectional study in urban western Iran

**DOI:** 10.1186/s12889-021-10261-6

**Published:** 2021-01-28

**Authors:** Nader Rajabi Gilan, Mehdi khezeli, Shirin Zardoshtian

**Affiliations:** 1 Social Development & Health Promotion Research Center, Health Institute, Kermanshah University of Medical Sciences, Kermanshah, Iran; 2 Department of Sport Management, Faculty of Sport Science, Razi University, Kermanshah, Iran

**Keywords:** Life satisfaction, Self-rated health, Socioeconomic status, Social trust, Well-being

## Abstract

**Background:**

Life satisfaction is an important component in designing strategies to improve health outcomes in different groups of society. This study aimed to investigate the effect of subjective socioeconomic status (SSS), social capital (SC), self-rated health (SRH), and physical activity (PA) on life satisfaction (LS) in Iran.

**Methods:**

This cross-sectional study was conducted on 1187 people (643 men and 544 women) lived in five western cities in Iran. The sampling method was multistage clustering. Data collection tool was a five part questionnaire including demographic characteristics, socioeconomic status ladder, social capital scale, a question to measure physical activity, and the life satisfaction scale. Data were analyzed using independent t-test, one way ANOVA, and Ordinal Logistic Regression.

**Result:**

Life satisfaction was higher in married men and women compared to single and widows (*p* < 0.05). Among the variables included in the main model, the significant predictors were college education (− 0.500), marriage (coefficient = 0.422), age 25–34 years (coefficient = − 0.384), SRH (coefficient = 0.477), male sex (coefficient = 0.425), SSS (coefficient = 0.373), trust (coefficient = 0.115), and belonging and empathy (coefficient = 0.064).

**Conclusion:**

SRH and SSS were significant predictors of life satisfaction in west Iranian society. Being married was associated with higher LS, but college education affects LS adversely.

## Background

Well-being assessment is one of the main priorities for governments, international organizations, health companies and research institutions [1]. Life satisfaction (LS) as a main component of subjective well-being is routinely used as an only measurement of subjective well-being in many studies [2, 3]. LS is a universal assessment of one’s life based on one’s subjective criteria. it includes an individual’s self-assessment about life’s adaptation according to the internal expectations [4]. The study of LS given that it depends on individuals and social context can be examined at different times and various demographic groups [5]. Researchers have shown that people with high level of LS compared to those with lower level tend to have more positive social relationships, marital satisfaction, and experience social support [6]. In the present study according to the previous studies and literature, we examined the association between some demographic, physical and social factors with LS. Some studies emphasized that four factors directly influences the level of LS including physical health condition, mental health condition, social relationship and environment [7].Self-rated health (SRH) represents a meaningful, subjective indicator for health status. It is an interested new assessment based on the individual appraisal of one’s health that includes an overall sense of functioning, physical, mental and social dimensions of health [8]. The self-rated health index has been widely used in recent years in studies and the results have shown that it is associated with life satisfaction [9–11].

Physical activity is another variable related to well-being and life satisfaction which refers any movement in daily life and has three levels of work, sport and leisure [12]. Worldwide, physical inactivity is one of the leading causes of disease, disability, and preventable death, while PA is an important protective factor against the development of chronic diseases [13]. Although a mass body of studies and evidence show that physical activity is directly related to various aspects of health [14, 15], there is less evidence to link physical activity to life satisfaction and mental well-being based on community-based studies. In this regard, some studies in different groups confirm the positive relationship between PA and LS, and mental well-being [16–18], instead, some studies have reported a lack of significant relationship or contradictory results [19, 20].

One of the most important limitations of some studies in the field of LS is ignoring social relationships and factors [21]. Social capital (SC) is one of the main social factors in sociology, health promotion, and health economic literature [22]. SC defined by Putnam and determined by variables such as social trust, cross-trade norms, and social network density [23]. Results from a study in China indicated that social capital had the significant positive effect on LS, according to which the social capital improves the life satisfaction through promoting positive affect [24]. Another study with community based approach in Rotterdam, Netherlands showed the significant positive association between neighborhood-based social capital and individual LS [25].

There are several definitions and measures for recognizing social capital, introduced by Asadullah et al. (2017), in which the average response to the trust question are acknowledge as the standard measure for social capital [26]. One of the approaches to the social capital is to divide it into bonding and bridging types [27]. Bonding social capital refers to robust social ties based on family relationships, kinship, gender, ethnicity, and religion. Bridging social capital is also based on the relationships of individuals and groups with dissimilar others [28]. Social capital in traditional societies almost includes bonding aspect with specific social trust, and in modern societies is mostly a bridging aspect with generalized social trust [28]. Also, the cultural context of Iran, especially in the western provinces where this research has been conducted is socially unbalanced that create a mixture of traditional and modern social capital.

Socioeconomic status (SES) is another important social variable affecting subjective well-being examined in this study. SES is one of the strongest predictors of individual diseases and mortality [29], that is why research on the role of socioeconomic factors on health and disease has been increasing worldwide in recent years [30]. Based on the evidence and studies, Huang et al. have defined two main types of socioeconomic status. Objective SES is the economic and social position about others, which is widely measured using three indicators: income, education, and occupation. In contrast, subjective SES is a person’s conception of his or her position compared with that of others [31]. There are many qualitative and quantitative studies show that high levels of socioeconomic status are associated with higher levels of life satisfaction in different social groups [32–34].

Despite the importance of social factors and PA on life satisfaction, we did not find a study in Iran on this issue in the general population. Therefore, this study aimed to investigate the relationship between SRH, SC, SSS, and PA with LS in five western provinces of Iran. Main research hypothesis of this study was that socioeconomic status, social capital and physical activity are related to and predict the life satisfaction in general population of urban western Iran.

## Method

### Study design and participants

This was a cross-sectional study. The target population of the study was the population aged above 16 years nearly 2 million people in five province centers located at west of Iran include Kermanshah, Sanandaj, Ilam, Hamadan, and Khorramabad. Sample size was calculated using the results of the similar previous studies, according to which the final sample size was 1268. The proportion of each city (Kermanshah 483 people, Hamadan 283, Sanandaj 215, Khorramabad 187 and Ilam 100) was calculated according to the each urban population and cluster sampling method was used to select the samples. For this purpose, each municipality district was considered as a cluster in each city. The number of municipality districts or clusters included: Kermanshah eight districts, Hamadan four districts, Sanandaj three districts, Khorramabad three districts, and two districts. Then, two neighborhoods were randomly assigned from each cluster. Finally, according to the population of the neighborhoods, samples were selected in each neighborhood using convenience sampling method. Inclusion criteria were consent to participate in the study, lack of physical disability and acute mental illness, and age over 16 years. In the present study, 1268 individual received the study questionnaire and finally 1187 participants completed the questionnaire which showed the response rate of 93.5%.

### Measurements

Data collection tool was a five part questionnaire. The first section was questions on demographic information including age, sex (1- male; 2- female), marital status (1- single; 2- married; 3- widow), and education levels (1- under diploma; 2- diploma; 3- college). PA in second part was assessed with one question as “how many days a week have you had at least 30 minutes of moderate to vigorous physical activity such as walking, swimming, fitness, mountaineering, etc.? “ on a four point scale (1- never; 2- one to three time a month; 3- one to two times a week; 4- three and above times a week) [35]. The third part of questionnaire was a SSS scale which assesses current SSS using a social ladder [36, 37]. The subjective evaluation of SES is the self-conceiving of the individual’s position in the social structure. This scale assesses perception of individuals about job, education, and wealth dimensions on a 10-point ladder, in which the higher score indicated the better perception about SSS.

The fourth part was the Satisfaction with Life Scale (SWLS) developed by Diener et al. (1985) [4]. This scale consists of five-items including: 1) In most ways my life is close to my ideal, 2) The conditions of my life are excellent, 3) I am satisfied with my life, 4) So far I have gotten the important things I want in life, 5) If I could live my life over, I would change almost nothing. Each item rates on a five-point Likert scale (1 = strongly disagree to 5 = strongly agree), and total score of scale can be ranged from 5 to 25, according to which higher scores indicates greater LS. According to the previous studies we divided scores into 5 categories from very dissatisfied to very satisfied [10]. Then due to the small number of respondents who assessed their LS as very dissatisfied and very satisfied, these two categories are combined in the dissatisfied and satisfied categories, and finally we had three categories including: very dissatisfied or dissatisfied / so-so / satisfied or very satisfied. Finally 3-point scale for each item was recorded, with the total scale range from 5 to 15. This tool has been validated in an Iranian research by Vahedi and Eskandari (2010) with an acceptable reliability using Cronbach’s alpha. Concurrent validity of the Life Satisfaction Scale was assessed through correlation with WHO Quality of Life Questionnaire, according to which there were significant correlations with the four subscales of QOL questionnaire including mental health, physical health, social relationships, and environmental health [38].

The fifth part of the questionnaire assessed the self-rated health using a single question; “How is your health in general?” extracted from the World Health Organization Quality of Life Questionnaire (WHOQOL-BREF). This question measures the SRH on a 5-point Likert scale (very bad / bad / not good not bad / good / very good). The higher score shows the better SRH [39].

The final part of the questionnaire was Social Capital scale introduced by Rafiey et al., (2019) consists of 20 items on five point Likert scale that measure two forms of bonding and bridging SC. The total score of scale in continuous measuring can be ranged between 20 and 100 score in which higher score indicated the higher level of SC (Table 1). In the present study we used the bonding social capital subscale consisted of three factors including empathy and belonging (six question), trust (three question), and partnership (two question) [28]. The reliability of the questionnaire was confirmed with Cronbach’s alpha coefficient of 0.92.

**Table 1:** definition, measure (Scale/ question) and range score of main variables in this study

variable	Definition	Scale/ question		Range of scores	
				Min	max
SSS	Subjective Socioeconomic Status. A self-assessment by means of early-life education, longest occupation, and late-life financial conditions.	subjective socioeconomic status was assessed with a single question on a 10-part ladder scale Question Please view a drawing ladder with 10 rungs, represents where people stand in society. At the top of the ladder are the people who have the most money, most education, and best jobs. At the bottom are the people who have the least money, least education, worst jobs, or no job. Please place an ‘X’ on the rung that best represents where you think you stand on the ladder.”		1	10
PA	Physical activity (PA) which refers any movement in daily life	PA was assessed with a five-point Likert question: “How often do you participate in sports or physical exercise? “Never”, “1–3 time a month”, “1–2 days a week”, “three and above time in week”.		1	4
LS	Life Satisfaction is a universal assessment of one’s life based on one’s subjective criteria. This criterion is individual’s self-assessment about life’s adaptation according to the internal expectations	**scale** LS was assessed by Diner et al. (1985) scale with five-item; each rated on a five-point Likert scale (1 = strongly disagree to 5 = strongly agree). Total score can be ranged on a five-point scale—very dissatisfied (1) to very satisfied (5). Due to the small number of respondents who assessed their LS as “very dissatisfied” and “satisfied”, these two categories are combined in the Dissatisfied and Satisfied categories, and finally we have three categories including: Very dissatisfied or dissatisfied / so-so / satisfied or very satisfied.		5	15
SRH	individual appraisal of one’s health that includes an overall sense of functioning, physical, mental and social dimensions of health	A single question; extracted from the World Health Organization Quality of Life Questionnaire (WHOQOL-BREF). “How is your health in general?” This question measures the SRH on a 5-point Likert scale (very bad / bad / not good not bad / good / very good).		1	5
SC	Social Capital is defined as trust, norms, and networks that make cooperation and collaboration easy to achieve.	**Scale** SC was assessed by Rafiey et al. (2016) scale consists of 20 items on five point Likert scale that measure “SC” in two dimensions of bonding and bridging. In this study we analyzed three factors related bonding social capital including empathy and belonging (six question), trust (three question), and partnership (two question	**empathy and belonging**	6	30
			**trust**	3	15
			**partnership**	2	10
			**SC total**	11	55

### Ethical statement

This study received the ethics approval from the Research Ethics Committee of Kermanshah University of Medical Sciences (No.IR.KUMS.REC.1398.118). Written informed consent form was obtained from all of the participants.

### Data collection and statistical analysis

Data were collected from October to November 2019 by trained and informed interviewers about the environment and social context of each district. Data were analyzed by Statistical Package for the Social Sciences **(**SPSS-18) and STATA-8. Since, our dependent variable (LS) was an ordinal variable; hence the appropriate technique for the analysis was Ordinal Logistic Regression Method [10, 40]. Also we used independent t-test, and one way ANOVA to compare the mean of LS in different groups. Mean and standard deviation was used to report the descriptive status of variables. A 95% level of confidence was assumed.

## Results

In the present study, mean age of the participants was 33.12 ± 12.61 years. More than half of the participants were male (54.2%); had college education (51.3%), and were single (50.6%). Also the highest frequency in age groups was in 25–34 years with 33.2%.

Results also showed that 330 (27.8%) participants reported that they were very dissatisfied or dissatisfied with life, 464 (39.1%) neither dissatisfied nor satisfied, and 393 (33.1%) were satisfied or very satisfied with life. As shown in Table 2, the mean score of life satisfaction in women was higher than men (*p*-value < 0.001). Mean score of LS also was different among SRH groups (*p*-value < 0.001), and SSS categories (*p*-value < 0.001). Results provided in Table 3 showed that the life satisfaction was significantly higher in married (both genders) than single and widows, while singles and widows were not significantly different.

**Table 2:** mean (SD) of LS according to the independent variables (*n* = 1187)

Independent variables	Category	N [%]	Life satisfaction (LS)		***P***-value
			Mean	(SD)	
**Sex**	***Male***	643 [54.2]	8.83	(2.88)	0.001
	***Female***	544[45.8]	9.43	(3.29)	
**Age group**	***16–24 year***	360 [30.3]	9.10	(2.95)	0.151
	***25–34 year***	394[33.2]	8.90	(3.15)	
	***35–44 year***	220 [18.5]	9.10	(2.89)	
	***45–54 year***	118[9.9]	9.33	(3.14)	
	***55–64 year***	69 [5.8]	9.60	(3.71)	
	***65–75 year***	26[2.2]	10.30	(3.44)	
**Marriage**	***single***	601 [50.6]	8.72	(2.92)	0.001
	***Married***	542[45.7]	9.62	(3.17)	
	***Widow and divorced***	44 [3.7]	8.06	(3.32)	
**Education**	***Under diploma***	190 [16.0]	9.12	(3.27)	0.629
	***diploma***	388[32.7]	8.99	(3.02)	
	***College***	609 [51.3]	9.18	(3.08)	
**physical activity (PA)**	***Never***	276 [23.3]	8.21	(3.00)	0.001
	***1–3 times a month***	316[26.7]	8.85	(2.99)	
	***1–2 times a week***	364 [30.7]	9.74	(3.15)	
	***3 or more times a week***	231 [19.5]	9.54	(2.94)	
**SRH**	***Very bad***	59 [5.0]	7.54	(3.35)	0.001
	***Bad***	87[7.3]	7.25	(2.89)	
	***Not bad – not good***	318 [26.8]	8.14	(2.82)	
	***Good***	547 [46.1]	9.61	(2.92)	
	***Very good***	176 [14.6]	10.67	(2.85)	
SSS	***1***	81 [6.8]	6.61	(2.22)	0.001
	***2***	99[8.3]	7.47	(2.98)	
	***3***	148 [12.5]	7.79	(2.80)	
	***4***	152 [12.7]	8.18	(2.52)	
	***5***	275 [23.1]	9.21	(2.65)	
	***6***	152 [12.8]	9.43	(2.83)	
	***7***	130[11.0]	10.50	(2.63)	
	***8***	93 [7.8]	11.46	(2.85)	
	***9***	30 [2.5]	13.73	(1.52)	
	***10***	27 [2.3]	12.22	(2.96)	
	**Total participants**	1187[100]	9.11	(3.05)

**Table 3:** Comparison of average life satisfaction score by gender

Gender	Mean (SD) of LS			F	***P***-value	Tukey test
	Single	Married	Widow			
**Male**	8.44(2.81)	9.39(2.88)	5.80(1.78)	11.57	0.001	Married >Single, widow
**Female**	9.14(3.04)	9.87(3.44)	8.35(3.38)	5.42	0.005	Married >Single, widow
**Total**	8.72(2.92)	9.62(3.17)	8.06(3.32)	14.94	0.001	Married >Single, widow

Ordinal Logistic Regression was used to determine the factors affecting life satisfaction. We had two analysis models: one without SRH (model 1), and another includes SRH (model 2). Estimates were slightly different in the two models: in model 1, physical activity 1 to 2 times a week was significant, but model 2 showed that the role of physical activity was not significant.Table 4 shows the main determinants of life satisfaction in two model according to the Ordinal Logistic Regression. Results of model 2 showed that college education (− 0.500, *p*-value = 0.008) was a significant predictor to lower LS compared to under diploma education, while diploma and under diploma education were no different. Married condition (coefficient = 0.422, *p*-value = 0.008) was significant predictor compared to single and widowed. Another predictors were male (coefficient = 0.425, *p*-value = 0.005), age of 25–34 years compared to lower age (coefficient = − 0.0384, *p*-value = 0.008), SRH (coefficient = 0.477, *p*-value < 0.001), and SSS (coefficient = 0.373, *p*-value < 0.001). Among three dimension of bonding SC, trust (coefficient = 0.115, *p*-value < 0.001), and belonging and empathy (coefficient = 0.064, *p*-value < 0.001) were significant while partnership was not significant. Residence and physical activity were not significant.

**Table 4:** results of Ordinal Logistic Regression for determinants of LS (*n* = 1187)

Variables	Categories	Model 1			Model 2		
		Coef(β)	SE	***p***-value	Coef(β)	SE	***p***-value
**Education**	Under diploma	**Ref**			**Ref**		
	*diploma*	−0.025	0.193	0.893	−0.041	0.195	0.830
	*College*	**−0.408**	**0.186**	**0.028**	**−0.500**	**0.189**	**0.008**
**Marital status**	*Single*	**Ref**			**Ref**		
	*Married*	**0.383**	**0.156**	**0.015**	**0.422**	**0.158**	**0.008**
	*Widow*	0.025	0.358	0.944	**0.170**	**0.363**	**0.639**
**Sex**	*Female*	**Ref**			**Ref**		
	*Male*	**0.368**	**0.128**	**0.004**	**0.425**	**0.129**	**0.001**
**Age group**	16-24 years	**Ref**			**Ref**		
	*25–34 years*	**− 0.413**	**0.171**	**0.016**	**− 0.384**	**0.172**	**0.025**
	*35–44 years*	−0.267	0.215	0.208	−0.135	0.215	0.529
	*45–54 years*	−0.376	0.261	0.150	−0.242	0.265	0.361
	*55–64 years*	−0.140	0.319	0.660	0.012	0.321	0.970
	*65–75 years*	0.592	0.481	0.218	0.801	0.493	0.104
**Residence**	*Sanandaj*	**Ref**			**Ref**		
	*Hamadan*	−0.095	0.202	0.635	−0.213	0.206	0.300
	*Ilam*	0.547	0.266	0.040	0.448	0.267	0.094
	*Khorram Abad*	0.330	0.222	0.136	0.307	0.224	0.171
	*Kermanshah*	−0.128	0.182	0.482	−0.322	0.187	0.086
**physical activity**	*never*	**Ref**			**Ref**		
	*1–3 times a month*	0.179	0.177	0.312	0.088	0.179	0.620
	*1–2 times a week*	**0.359**	**0.175**	**0.040**	0.235	0.177	0.185
	*three and above days in week*	0.196	0.197	0.320	−0.013	0.201	0.945
**Belonging and Empathy**		**0.075**	**0.015**	**0.001**	**0.064**	**0.015**	**0.001**
**Trust**		**0.123**	**0.026**	**0.001**	**0.115**	**0.026**	**0.001**
**partnership**		0.091	0.037	0.016	0.060	0.038	0.115
**SSS**		**0.411**	**0.033**	**0.001**	**0.373**	**0.033**	**0.001**
**SRH**		**–**	**–**	**–**	**0.477**	**0.076**	**0.001**
**Log likelihood**		**− 964.14**			**−944.38**		
**LR chi2**		**(21) 447.63**			**(22) 487.41**		
**Prob > chi2**		**0.001**			**0.001**		
**Pseudo R2**		**0.1885**			**0.2051**		
**Number of obs**		**1171**			**1171**		

## Discussion

This study investigated the associations between SSS, SRH, bonding SC, and PA with LS in urban population of western Iran.

The results of this study showed that SSS have a significant effect on life satisfaction. Ng and colleagues also showed that better perceived condition had a positive and significant effect on LS [10]. The results of a study in China showed that subjective well-being was affected by socioeconomic status, and the most influential variables in this regard were education, employment and income [41]. A study in Russia and Ukraine by Abbott & Sapsford (2006) showed that human capital, financial conditions (economic status and ability to provide the necessities), income satisfaction, and family facilities had direct relationships with LS [42]. Also a study by Asadullah and a Chaudhury (2012) in Bangladesh showed that higher objective income and economic condition is related with the higher level of life satisfaction [43]. Here we can refer to Easterlin’s theory of income and subjective well-being. Easterlin believes that increasing personal income can be a source of LS and happiness which confirms the results of the present study. However, the second part of Easterlin’s theory, which is considered as a theoretical paradox and has shown that the economic growth of society does not cause happiness and mental well-being, has not been studied in this study [44]. It seems that when people compare their income and economic situation with others and are placed in a lower situation, are likely to experience pessimism and hopelessness [45]. This can reduce satisfaction from health, occupation, and income, and subsequently can lead to reduced LS and happiness. If one’s assessment of living conditions be negative, this can influences and evolves many of the behaviors and attitudes toward the individual’s situation and social circumstances [46].

Other result of this study showed a positive and significant relationship between bonding SC and LS. This finding was consistent with study by Ghasemi et al. (2017) on the relationship of social capital with LS in Iran [47]. Woo (2017) in a study in South Korea and Taiwan showed that social capital was positively correlated with LS in the Taiwanese sample by controlling variables such as subjective social status, self-rated health, gender, and individualism tendency while there was no significant relationship between SC and LS in the South Korean sample. The South Korean case revealed that social capital is not a good predictor for LS in an environment where success is over-emphasized [48].

Regression analysis showed that among bonding SC dimension, belonging and empathy, and trust had the direct and meaningful effects on LS. Mironova (2015) assessed the relationship between different types of trust (institutional, public, and social) with LS, according to which structural equation model showed that social trust had the most direct effect on LS [49], consistent with the present study. Trust is the heart of social and political stability in society and is important for the health and interpersonal relationships, so that the decline of trust in any society is a major constraint on social economic development [49]. Trust is a major factor in social well-being and one of the most important indicators of social well-being, and assessment of individuals’ mental life satisfaction.

The results of the present study indicated the positive and significant effect of SRH on LS. Results of a study in Europe indicated that there was a positive association between self-reported health status and LS across countries [50]. In another study SRH was negatively associated to depressed mood, mediated by life satisfaction. An interactions showed that better economic situation compensated the effect of a low SRH on life satisfaction [51]. Schneider et al. also pointed out in another direction that life satisfaction is related to self-evaluation of health in the elderly [52]. However, the literature shows that there is a positive relationship between SRH and LS, and the present study concluded that higher SRH scores predict higher LS.

Other result of this study showed that college education level had a negative and decreasing effect on LS. This finding is inconsistent with the results of Jiménez’s (2011) [53] and Martikainen (2010) [54]. However, results of a study in Europe showed that one level increase in education degree leads to an average of 0.03 decline in LS scores [55]. It seems that the negative impact of higher education on the LS in the present study is due to the high unemployment rate of university graduates in Iranian society [56]. As noted by Martikainen (2010), job satisfaction is a main influencing factors on life satisfaction [54]. It can also be said that people with higher education experience more stress and strain, which can reduce their life satisfaction.

The results showed that LS scores were significantly higher among married people than single and widows. Marriage in the regression model had a positive and significant effect on LS which was consistent with similar studies [57, 58]. Although the mean score of LS in women was higher than men, but more detailed results revealed that LS score in male and female married was not different and significantly was higher than singles and widows. Also, singles and widows in both sexes had no different LS score. It seems that regardless of being male or female, marriage is an appropriate emotional shield to promote mental health and well-being in individuals. Marriage provides various consequences and incentives such as lower mortality risk, shared household goods, and other benefits [59]. Stutzer & Frey (2006) argue that marriage and individual well-being are positively correlated, because marriage is an additional source of self-esteem and married people are less likely to experience loneliness and have the opportunity to benefit from a supportive relationship [60].

Self-reporting was one of the limitation of this study which can increase the likelihood of over or under reporting. Another limitation of this study was using the subjective SES scale to assess the socioeconomic situation and not to examine the participants’ objective income and its relationship with LS. An accurate assessment of income level in cross-sectional studies in Iran is difficult due to the lack of transparency, and the respondents’ refusal to report their actual income. It should be noted that the perception of existing income is one of the aspects of subjective SES ladder that has been studied in this study. However, the present study was conducted with an appropriate sample size in urban area of five western provinces of Iran, which can provide a proper assessment of the life satisfaction of women in the urban population of western Iran.

## Conclusion

This study showed that better SSS and being married had an increasing effect on LS in Iranian society. Belonging and empathy, and trust as emotional and cognitive dimensions of SC also increased the LS. It seems that social policies that create trust at the micro and macro level, and providing proper mechanisms for marriage in appropriate age groups can be useful in improving LS with considering the cultural and social contexts of western Iran.

## Data Availability

The data sets used and analyzed in this study are available from the corresponding author on reasonable request.

## Abbreviations

PA: Physical Activity
LS: Life Satisfaction
SC: Social Capital
SRH: Self-rated Health
SES: Socioeconomic Status
SSS: Subjective Socioeconomic Status
